# Three new species of frogs of the genus *Pristimantis* (Anura, Strabomantidae) with a redefinition of the *P. lacrimosus* species group

**DOI:** 10.3897/zookeys.993.53559

**Published:** 2020-11-16

**Authors:** Santiago R. Ron, Julio Carrión, Marcel A. Caminer, Yerka Sagredo, María J. Navarrete, Jhael A. Ortega, Andrea Varela-Jaramillo, Gabriela A. Maldonado-Castro, Claudia Terán

**Affiliations:** 1 Museo de Zoología, Escuela de Biología, Pontificia Universidad Católica del Ecuador, Av. 12 de Octubre y Roca, Aptdo. 17-01-2184, Quito, Ecuador Pontificia Universidad Católica del Ecuador Quito Ecuador

**Keywords:** Amazon, Andes, Brachycephaloidea, morphology, phalanges, phylogeny, *Pristimantis amaguanae* sp. nov., *Pristimantis nankints* sp. nov., *Pristimantis romeroae* sp. nov., subarticular tubercles, systematics, taxonomy, Terrarana

## Abstract

A new phylogeny for the *Pristimantis lacrimosus* species group is presented, its species content reviewed, and three new species described from the eastern slopes of the Ecuadorian Andes. Our phylogeny includes, for the first time, samples of *P. aureolineatus*, *P. bromeliaceus*, and *P. lacrimosus*. The morphology of hyperdistal subarticular tubercles is also assessed among 21 species of *Pristimantis*. The *P. lacrimosus* species group is composed of 36 species distributed in the Chocó, Guiana, and Amazon regions of tropical South America with a single species reaching Central America. Ancestral area reconstruction indicates that, despite its high diversity in the Amazon region, the *P. lacrimosus* group originated in the Pacific basin, Chocó region of Ecuador and Colombia. *Pristimantis amaguanae***sp. nov.** is most closely related to *P. bromeliaceus*. It differs from *P. bromeliaceus* by being smaller, having transversal dark bands in the hindlimbs (absent or faint in *P. bromeliaceus*) and the absence of discoidal fold (present in *P. bromeliaceus*). *Pristimantis nankints***sp. nov.** and *P. romeroae***sp. nov.** are part of a clade of predominantly light-green frogs that includes *P. acuminatus*, *P. enigmaticus*, *P. limoncochensis*, and *P. omeviridis*. *Pristimantis nankints***sp. nov.** and *P. romeroae***sp. nov.** can be distinguished from all of them by the presence of a dark dorsolateral stripe that borders a light green band on a green background. Hyperdistal tubercles are present in all examined species of the *P. lacrimosus* species group and its sister clade. Species with hyperdistal tubercles are characterized by having relatively long terminal phalanges and narrow T-shaped expansion at the end of the terminal phalange. We discuss the phylogenetic distribution of these characters and their potential diagnostic significance.

## Introduction

With 540 species *Pristimantis* is the most diverse vertebrate genus and represents a majority of the formally described anuran diversity in the tropical Andes of Colombia and Ecuador (AmphibiaWeb 2019). Moreover, molecular studies are revealing a high proportion of cryptic diversity (e.g., Elmer et al. 2007; Ortega-Andrade et al. 2015; Rivera-Correa and Daza 2016). Molecular-based systematic reviews should substantially increase the number of described *Pristimantis* species in decades to come.

One phenotypically distinctive group of *Pristimantis* is the *P. lacrimosus* group. Species of this group are frequently found in bromeliad plants and have broad and flattened heads with acuminate snouts (Hedges et al. 2008). The group was first recognized by Lynch and Duellman (1980) as an assemblage of species within the *Pristimantis unistrigatus* species group. It contained *P. bromeliaceus*, *P. lacrimosus*, *P. mendax*, and *P. petersi*. Subsequent reviews added more species (e.g., Guayasamin et al. 2006; Lynch 1980) until reaching 18 species by 2008 (Hedges et al. 2008) and 25 by 2014 (Padial et al. 2014). Rivera-Correa and Daza (2016) presented a molecular phylogeny showing that the group was paraphyletic because it formed two clades that were not sister to each other. One clade (named “A”) included three Colombian species, the other, “clade B”, included the remaining species of the *P. lacrimosus* group. Because the phylogenetic position of *P. lacrimosus* is unknown, they refrained from defining which of the two clades should be considered the *P. lacrimosus* species group. Nevertheless, Gonzalez-Duran et al. (2017) created a new species group for clade A, the *P. boulengeri* group. They hypothesized that clade B corresponds to the *P. lacrimosus* species group based on a figure of the neotype of *P. lacrimosus* (Lynch and Schwartz 1971) showing the absence of double distal subarticular tubercles on fingers III or IV, a diagnostic character for the *P. boulengeri* group.

During fieldwork in cloud forests of the Amazon Basin of Ecuador, field teams from Museo de Zoología at Catholic University of Ecuador found three distinctive species that belong to the *Pristimantis lacrimosus* species group. Herein we describe them and present a new phylogeny for the group including, for the first time, *P. aureolineatus*, *P. bromeliaceus*, and *P. lacrimosus*.

## Materials and methods

### Morphology

The format for the descriptions follows Lynch and Duellman (1997). The terminology and definition of diagnostic characters follows Duellman and Lehr (2009). Specimens were preserved in 10% formalin and stored in 70% ethanol. Sex was determined by examining the presence of vocal slits and gonadal inspection. Measurements were taken with digital calipers (to nearest 0.01 mm) following the methodology described by Navarrete et al. (2016). Examined specimens belong to the herpetological collection at Museo de Zoología, Pontificia Universidad Católica del Ecuador, Quito (**QCAZ**), and are listed in Appendix 1.

**Figure 1.**
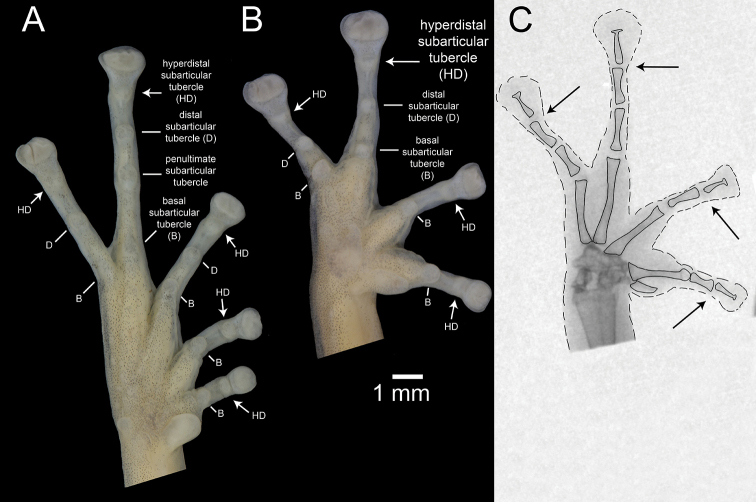
Palmar and plantar surfaces of *Pristimantis romeroae* sp. nov. and X-ray of the hand **A** ventral view of the left foot **B** ventral view of left hand **C** X-ray of the left hand. Holotype (QCAZ 41121). Arrows point to the hyperdistal subarticular tubercles. Abbreviations: B = basal, D = distal, HD = hyperdistal. In the x-ray image, dashed lines indicate the border of soft tissoes; continuous lines indicate edges of bones.

Fingers and toes are numbered preaxially to postaxially from I to IV and I to V, respectively. Relative lengths of toes III and V were determined by adpressing them against toe IV; lengths of fingers I and II were compared by appressing them to each other. While describing the hands and feet of the new species, we noticed the presence of an additional distal subarticular tubercle. These tubercles were recently named by Ospina-Sarria and Duellman (2019) as “hyperdistal”. We use the following terminology for the subarticular tubercles of *Pristimantis*, including hyperdistal tubercles (Fig. 1): (1) “basal” for the tubercles at the base of fingers and toes, (2) “penultimate” occurs only on toe IV and refers to the tubercle underlying the distal articulation of the first phalange, (3) “distal” for the tubercles underlying the proximal articulation of the penultimate phalange, and (4) “hyperdistal” for the tubercle underlying the articulation of the last phalange of each finger and toe. Except for the exclusion of hyperdistal tubercles, this terminology has been used in most systematic accounts of *Pristimantis* at least since Lynch and Duellman (1997) seminal publication (e.g., Duellman and Lehr 2007; Urgiles et al. 2019; Valencia et al. 2019). We recommend its use, as amended by Ospina-Sarria and Duellman (2019), for future terminological consistency.

**Figure 2.**
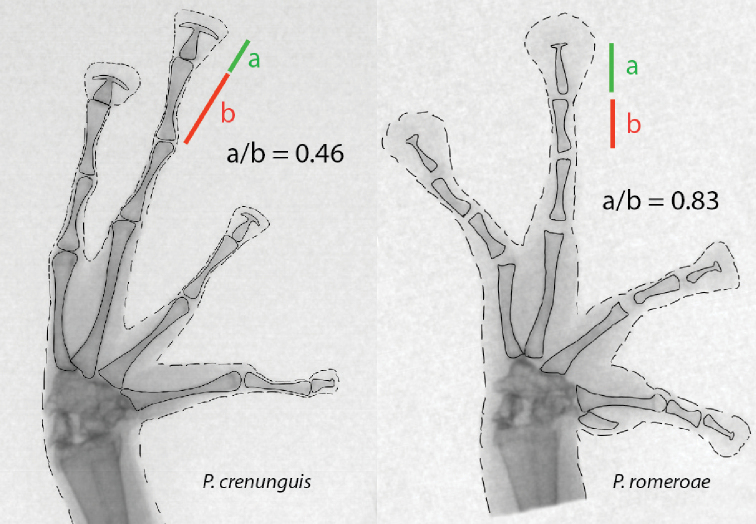
X-ray images of the left hand of adult *Pristimantis romeroae* sp. nov. and *P. crenunguis*. The ratios of the length of the ultimate and penultimate phalanges of finger III are shown. Dashed lines indicate the border of soft tissues; continuous lines indicate the edge of bones. *Pristimantis romeroae* sp. nov., QCAZ 41121, SVL = 31.1 mm; *P. crenunguis*, QCAZ 56506, SVL = 46.7 mm.

Subarticular tubercles underly articulations between phalanges (Lynch and Duellman 1997). To understand the osteological anatomy of the hyperdistal subarticular tubercles, we obtained X-ray images of 21 species of *Pristimantis* including two of the new species and three additional members of the *P. lacrimosus* species group (*P. acuminatus*, *P. enigmaticus*, and *P. limoncochensis*). The other species represent assorted lineages within *Pristimantis* (based on the phylogeny of Jetz and Pyron 2018). Digital X-ray images were obtained with the Thermo Kevex X-ray Imaging System at the QCAZ museum. To explore the relationship between phalange size and the presence of hyperdistal tubercles, we compared the length of the ultimate and penultimate phalanges of finger III (Fig. 2). *Pristimantis* have T-shaped terminal phalanges for which we measured the terminal expansion width in finger III. We measured the left hand except when fingers were twisted as result of fixation problems. If both hands had fixation problems, we excluded the specimen. All measurements were made with software ImageJ 2 version 1.52q (Rasband 2020).

### Phylogeny

We estimated the phylogenetic relationships of the new species based on DNA sequences of mitochondrial genes 12S rRNA (12S), 16S rRNA (16S), NADH dehydrogenase subunit 1 (ND1), their flanking tRNAs and the nuclear gene Recombination activating gene 1 (RAG-1). DNA was extracted from muscle or liver tissue preserved in 95% ethanol, using standard guanidine thiocyanate extraction protocols. We used a polymerase chain reaction (PCR) to amplify DNA fragments.

The primers used for the 12S amplification were obtained from Goebel et al. (1999), for 16S from Heinicke et al. (2007), for ND-1 from Zhang et al. (2013), Wiens et al. (2005), Moen and Wiens (2009) and Brito et al. (2017) and for RAG-1 from Hedges et al. (2008). PCR amplification was performed under standard protocols and sequenced by the Macrogen Sequencing Team (Macrogen Inc., Seoul, Korea).

Our matrix included 74 newly generated sequences of *Pristimantis* (Table 1). We also included congeneric sequences available on GenBank. To optimize taxon sampling, we blasted the 16S sequences of the new species to the GenBank database (blastn procedure). This search showed that the most similar sequences belong to species of the *Pristimantis lacrimosus* group. Therefore, we concluded that the new species are closely related to the *P. lacrimosus* species group clade B (*sensu*Rivera-Correa et al. 2016). We included all GenBank sequences of that clade as well as closely related species (based on Guayasamin et al. 2017) and representative samples of other species groups of *Pristimantis*. The GenBank sequence of specimen QCAZ 19664 (EU130579) was assigned to *P. acuminatus* by Elmer et al. (2007) but is updated to *P. omeviridis* based on Ortega-Andrade et al. (2015). For the outgroup we included several species of the subgenus Hypodictyon.

**Table 1.** GenBank accession numbers for DNA sequences used in the phylogenetic analyses.

Species	Voucher	12S	16S	RAG-1	ND1
*P. acerus*	KU 217786	EF493678.1	EF493678.1	NA	NA
	KU 217830	NA	EF493696.1	EF493432.1	NA
*P. altamazonicus*	KU 215460	EF493670.1	EF493670.1	NA	NA
***P. amaguanae* sp. nov.**	QCAZ 39274	MT636506	MT636529	MT635622	MT635661
*P. angustilineatus*	UVC 15828	NA	JN371034.1	NA	NA
*P. appendiculatus*	KU177637	EF493524.1	EF493524.1	NA	NA
*P. aureolineatus*	QCAZ 42286	MT636509	MT636530	MT635626	NA
*P. boulengeri*	MHUAA 8951	NA	KU724435.1	NA	NA
*P. brevifrons*	nrps 0059	JN991498.1	JN991433.1	NA	NA
*P. bromeliaceus*	QCAZ 16699	MT636505	MT636527	MT635618	MT635659
	QCAZ 62940	MT636512	MT636523	NA	MT635669
*P. calcarulatus*	KU 177658	EF493523.1	EF493523.1	NA	NA
*P. cedros*	MZUTI 1713	NA	KT210155.1	NA	NA
P. cf. mendax	MTD 45080	EU186659.1	EU186659.1	NA	NA
*P. crucifer*	KU 177733	EU186736.1	EU186718.1	NA	NA
*P. diadematus*	KU 221999	EU186668.1	EU186668.1	NA	NA
	KU179090	EF493522.1	EF493522.1	NA	NA
*P. dorsopictus*	MHUAA7638	KP082864.1	KP082874.1	NA	NA
*P. ecuadorensis*	CJ 5350	KX785339	KX785343	NA	KX785347
	CJ 5351	KX785340	KX785344	NA	KX785348
*P. enigmaticus*	QCAZ 40918	MT636513	MT636520	MT635636	MT635670
*P. galdi*	QCAZ 32368	EU186670.1	EU186670.1	EU186746	NA
*P. glandulosus*	KU 218002	EF493676.1	EF493676.1	NA	NA
*P. imitatrix*	KU 215476	EF493824.1	EF493667.1	NA	NA
*P. inusitatus*	KU 218015	EF493677.1	NA	NA	NA
*P. jaguensis*	MHUAA 7249	KP082862.1	KP082870.1	NA	NA
*P. jorgevelosai*	JDL 26123	NA	DQ195461.1	NA	NA
*P. lacrimosus*	QCAZ 55238	NA	MT636518	MT635629	MT635667
	QCAZ 59474	NA	MT636517	MT635633	NA
	QCAZ 40261	NA	MT636524	MT635623	MT635671
	QCAZ 59469	NA	MT636516	MT635632	NA
*P. limoncochensis*	QCAZ 43794	NA	MT636525	MT635627	MT635665
	QCAZ 19180	MN128395	MT636532	MT635620	NA
*P. melanogaster*		EF493826.1	EF493664.1	NA	NA
*P. mindo*	MZUTI 1382	NA	KF801584.1	NA	NA
	MZUTI 1381	NA	KF801583.1	NA	NA
	QCAZ 56512	NA	MT636522	MT635630	MT635668
	MZUTI 1756	NA	KF801581.1	NA	NA
	QCAZ 42197	MT636508	MT636531	MT635625	MT635664
*P. moro*	AJC 1860	JN991520.1	JN991454.1	JQ025191.1	NA
	AJC 1753	JN991519.1	JN991453.1	JQ025192.1	NA
***P. nankints* sp. nov.**	QCAZ 69137	NA	MT636514	MT635635	NA
*P. nyctophylax*	KU 177812	EF493526.1	EF493526.1	NA	NA
	QCAZ 32288	NA	MT636519	MT635621	MT635660
*P. omeviridis*	QCAZ 10564	MN128400	MK881398	MK881312	MT635658
	QCAZ 19664	NA	EU13057	MT635619	NA
*P. orcesi*	KU 218021	EF493679.1	EF493679.1	NA	NA
*P. ornatissimus*	MZUTI 4798	KU720464	KU720463	NA	KU720480
	MZUTI 4806	KX785337	KX785341	NA	KX785345
	MZUTI 4807	KX785338	KX785342	NA	KX785346
*P. pahuma*	MZUTI 493	NA	KT210158.1	NA	NA
*P. platydactylus*	MNCN 5524	FJ438811.1	EU192255.1	NA	NA
*P. pulvinatus*	KU 181015	EF186741.1	EF186723.1	NA	NA
*P. pycnodermis*	KU 218028	EF493680.1	EF493680.1	NA	NA
***P. romeroae* sp. nov.**	QCAZ 41121	MT636507	MT636528	MT635624	MT635662
*P. rubicundus*	QCAZ 58932	NA	MT372670	MT372613	NA
*P. schultei*	KU 212220	EF493681.1	EF493681.1	NA	NA
*P. subsigillatus*	KU 218147	EF493525.1	EF493525.1	NA	NA
	QCAZ 49637	NA	MT636521	MT635628	MT635666
	MECN 10117	NA	KF801580.1	NA	NA
*P. urani*	MHUAA 7471	NA	KU724442.1	NA	NA
*P. w-nigrum*	QCAZ 45200	MT636510	MT372703	MT372600	MT372569
	QCAZ 46256	NA	MT372704	MT372603	MT372571
	QCAZ 41818	NA	MT372691	NA	MT635663
*Pristimantis* sp.	ROM 43978	EU186678.1	EU186678.1	NA	NA
	KU 291702	EF493351.1	EF493351.1	NA	NA
	QCAZ 60398	NA	MT636515	MT635634	NA
	QCAZ 58956	MT636511	MT636526	MT635631	NA

The new sequences were assembled in Geneious 9.0 and then exported to Mesquite 3.61 (Maddison and Maddison 2019) where each genomic region was aligned using default parameters with Muscle 3.8 (Edgar 2004). Phylogenetic relationships were inferred for all genes concatenated using maximum likelihood (ML) as optimality criterion. The concatenated DNA matrix had 3974 bp and is available at http://zenodo.org under https://doi.org/10.5281/zenodo.4005737. Because different evolutionary processes have influenced each gene, we partitioned the data by gene and codon position to find the best model of evolution and partition scheme. To accomplish both tasks, we used the command MFP + MERGE (Chernomor et al. 2016; Kalyaanamoorthy et al. 2017) in software IQ-TREE multicore version 2.0 (Nguyen et al. 2015). To find the best phylogeny we run a search using IQ-TREE 2.0 under default settings. To assess branch support, we made 200 non-parametric bootstrap searches (-b 200 command) and 1000 replicates for the SH-like approximate likelihood ratio test (-alrt 1000 command; Guindon et al. 2010). We considered that branches with bootstrap values > 70 and SH-aLRT values > 80 had strong support. To calculate uncorrected *p*-distances of 16S we used MEGA 7.0 (Kumar et al. 2016).

### Biogeographic origin of the *P. lacrimosus* species group

Most species of the *Pristimantis lacrimosus* group occur in the Amazon and Pacific basins. To determine the biogeographic origin of the group, we carried out ancestral state reconstruction. Regions were coded as a binary character (Pacific or Amazon) and the reconstruction employed maximum likelihood as optimality criterion. The analysis was carried out in Mesquite 3.61 (Maddison and Maddison 2019) under the asymmetrical two parameter Markov-k model. The model finds the ancestral states that maximize the probability of the geographic distribution of the contemporary species given the phylogeny (see Pagel 1999 for a detailed description of the model). Ancestral states were traced using the best tree obtained from the maximum likelihood search (see above). Species distributed outside both basins (e.g., *P.* sp. ROM 43978, *P. jorgevelosai*) were coded as unknown because the two parameter Markov-k maximum likelihood model is binary and does not allow additional states. This restriction imposed by the Markov-k model was inconsequential to the results because only two species of the *P. lacrimosus* group (*P. moro* and *P.* sp.) were affected. Species distribution data was obtained from Anfibios del Ecuador (Ron et al. 2019), Lista de Anfibios de Colombia (Acosta-Galvis 2019), and the IUCN Red List website (https://www.iucnredlist.org/). River basin assignment was based on a digital map of South American basins obtained from the Food and Agriculture Organization GeoNetwork website (http://www.fao.org/geonetwork).

## Results

### Phylogeny and biogeography

**Figure 3.**
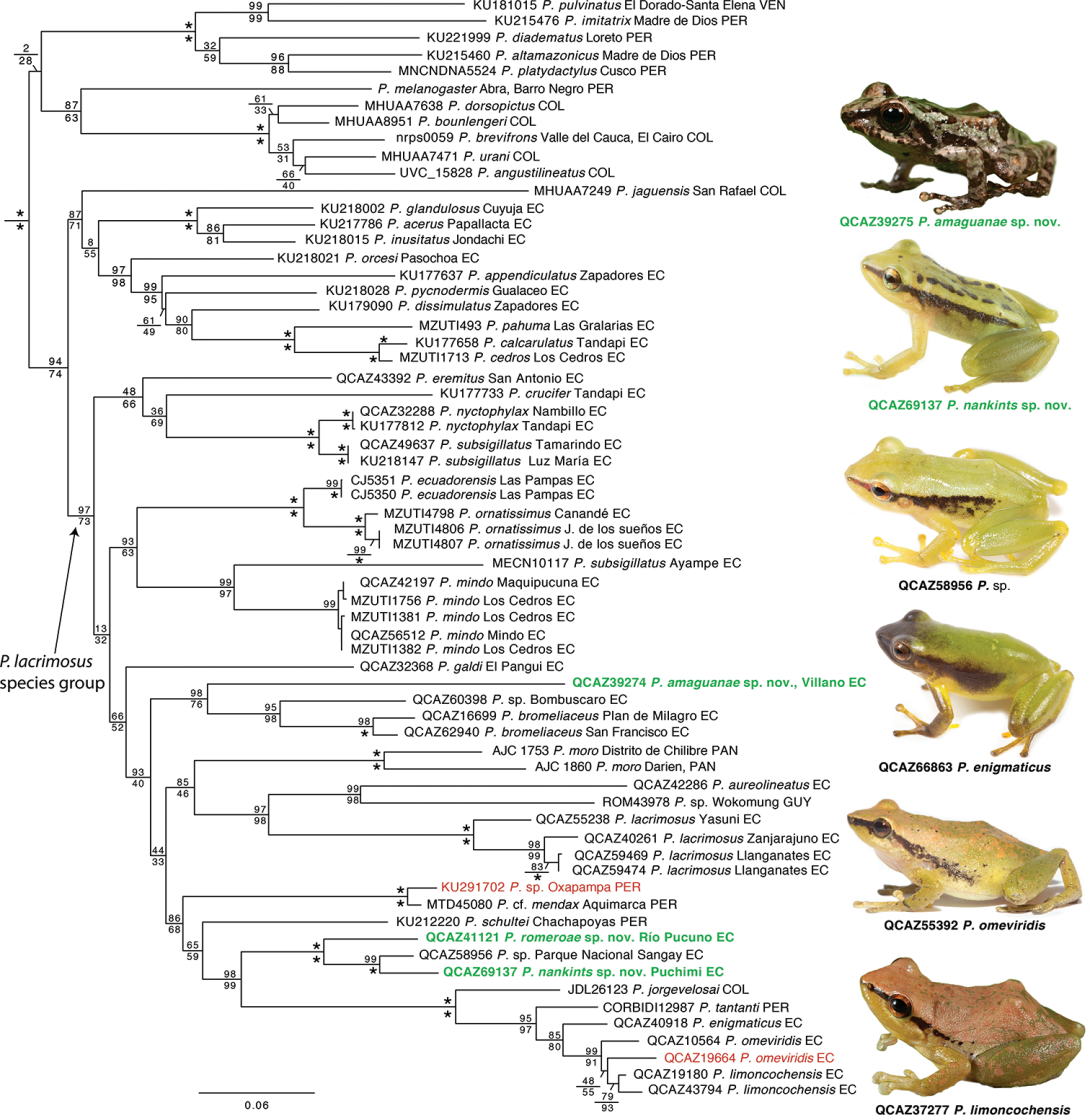
Phylogenetic relationships of the *Pristimantis lacrimosus* species group. Maximum likelihood tree for genes 12S, 16S, ND1 and RAG-1. SH-aLRT support (above) and non-parametric bootstrap support (below) are shown as percentages on branches. Asterisks indicate support values of 100 (bootstrap). Voucher number, species, and locality of the samples are shown next to each terminal; country is indicated by the following abbreviations: COL = Colombia, EC = Ecuador, GUY = Guyana, PAN = Panama, PER = Peru, VEN = Venezuela. The new species are shown with bold green characters. Changes in species identifications relative to the GenBank database are shown with red. Photographs show five species of the clade of green species and *P. amaguanae* sp. nov.. Outgroup is not shown.

The phylogeny (Fig. 3) shows strong support (SH-aLRT = 97, bootstrap = 73) for a clade that includes the species of the *Pristimantis lacrimosus* group (clade B, sensu Rivera-Correa et al. 2016) as well as *P. amaguanae* sp. nov., *P. crucifer*, *P. galdi*, *P. ecuadorensis*, *P. eremitus*, *P. enigmaticus*, *P. jorgevelosai*, *P. limoncochensis*, *P. nankints* sp. nov., *P. nyctophylax*, *P. omeviridis*, *P. ornatissimus*, *P. romeroae* sp. nov., and *P.* sp. Within this clade, the first two clades to diverge are distributed in the Pacific basin of Ecuador and include *P. crucifer*, *P. ecuadorensis*, *P. mindo*, *P. nyctophylax*, *P. ornatissimus*, and *P. subsigillatus*. The remaining species form a large clade distributed in the Amazon basin with the exception of *P. moro* (Central America), *P. jorgevelosai* (Magdalena River basin), and *P.* sp. (Guyana).

We also found strong support (SH-aLRT = 94, bootstrap = 74) for a sister clade relationship between the *P. lacrimosus* group (as redefined below) and a clade composed of 11 species including *P. appendiculatus*, *P. calcarulatus*, *P. cedros*, *P. jaguensis*, *P. pycnodermis*, and *P. orcesi*. Within the *P. lacrimosus* species group, *Pristimantis amaguanae* sp. nov. is most closely related to *P. bromeliaceus* and an undescribed species from Bombuscaro, Podocarpus National Park, Ecuador. Branch lengths in the phylogeny and its morphological distinctiveness indicate that *P. amaguanae* sp. nov. is evolutionarily independent from *P. bromeliaceus* and the undescribed species from Bombuscaro. Uncorrected *p*-genetic distances (gene 16S) between *P. amaguanae* sp. nov. and its sister clade range between 13.0 and 13.8%. *Pristimantis nankints* sp. nov. is sister to an undescribed species from Sardinayacu, Sangay National Park, Ecuador. Their genetic distance is 2.7%. Both species are sister to *P. romeroae* sp. nov. from Napo Province. They are separated by distances of 6.9 (*P.* sp.) and 7.0% (*P. nankints* sp. nov.) These three species are sister to a clade composed by *P. jorgevelosai*, *P. enigmaticus*, *P. limoncochensis*, *P. omeviridis*, and *P. tantanti*.

**Figure 4.**
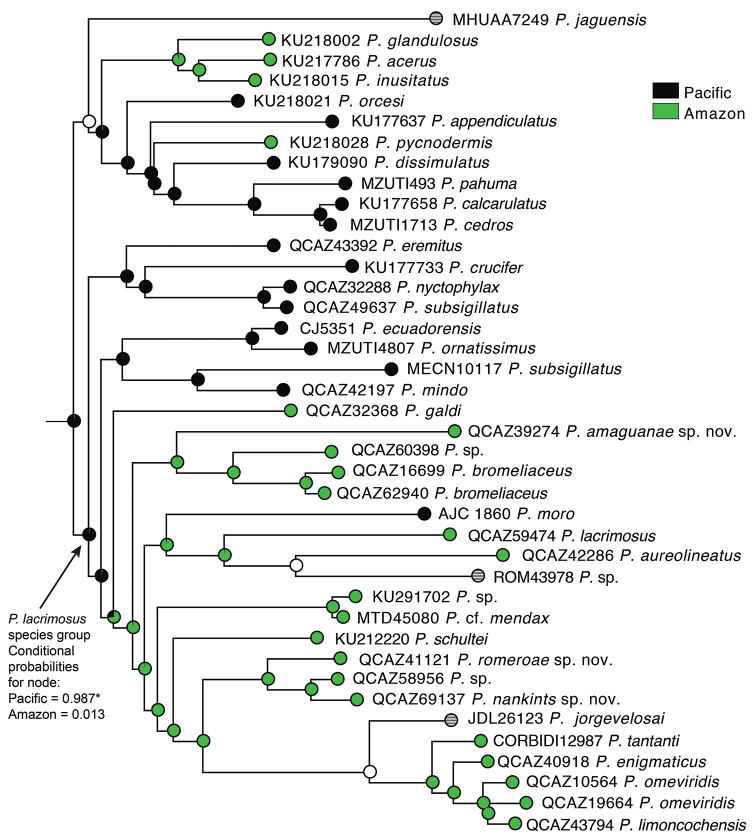
Ancestral reconstruction for geographic basin (Pacific vs. Amazon) in the *Pristimantis lacrimosus* species group. Reconstructions were based on maximum likelihood inference. Pie charts at nodes represent conditional probabilities for the Pacific (black) and Amazon (green) basins. The asterisk indicates significant support for the Pacific basin as ancestral area for the most recent common ancestor of the *P. lacrimosus* species group. White circles indicate equivocal state, striped circles indicate distribution outside the Pacific or Amazonian basins (coded as missing data).

The reconstruction of ancestral basin indicates that the group originated in the Pacific basin with a single colonization event to the Amazon basin (Fig. 4). There is one colonization event from the Amazon to Central America and the Pacific basin in *P. moro* and to the Río Magdalena basin (Colombia) in *P. jorgevelosai*.

### Hyperdistal subarticular tubercles and phalange morphology

Most examined species of *Pristimantis* lack hyperdistal tubercles (Table 2). However, the five species of the *P. lacrimosus* group have hyperdistal tubercles as well as *P. eriphus*, *P. katoptroides*, and *P. orcesi*. As expected, the hyperdistal tubercle is located in the articulation of the two terminal phalanges (Fig. 1). Among the 21 species of *Pristimantis*, the five examined species of the *P. lacrimosus* group are characterized by having: (1) long terminal phalanges, and (2) narrow T-shaped expansions in the terminal phalanges. Species with shorter terminal phalanges and long T-shaped expansions lack distinct hyperdistal tubercles (e.g., *P. crenunguis*, *P. condor*; Fig. 2). We found large variation in width of the T-shaped expansion in the terminal phalange. In some species the expansion is almost as long as the phalange (*P. crenunguis*) while in others it represents less than 1/3 of its length (*P. acuminatus*).

**Table 2.** Phalange morphometry and hyperdistal tubercle condition in several species of *Pristimantis*. Species of the *Pristimantis lacrimosus* species group are shown in bold. Species are ordered according to the relative length of the terminal phalanges (from low to high, terminal/penultimate). The five lowest T-width/terminal values are shown with italics. Note that species of the *P. lacrimosus* species group are characterized by having longer terminal phalanges, narrower T-expansions at the end of the terminal phalange, and hyperdistal tubercles. Hyperdistal tubercles were coded as “present” when they were similar in size to other hyperdistal tubercles in the same finger or toe.

QCAZ	Species	Terminal/ penultimate	T-width/ terminal	Hyperdistal tubercle
56506	*P. crenunguis*	0.463	0.980	absent
40057	*P. buckleyi*	0.467	0.597	absent
2308	*P. unistrigatus*	0.473	0.574	absent
63435	*P. bicantus*	0.485	0.578	absent
43313	*P. quinquagesimus*	0.535	0.921	absent
66850	*P. condor*	0.550	0.608	absent
39122	*P. lanthanites*	0.558	0.652	absent
26209	*P. appendiculatus*	0.574	0.538	absent
49633	*P. achatinus*	0.601	0.461	absent
61831	*P. pycnodermis*	0.626	0.455	absent
47731	*P. chomskyi*	0.670	0.583	absent
66559	*P. eriphus*	0.732	0.539	present
66881	*P. katoptroides*	0.743	0.495	present
39763	*P. orcesi*	0.752	0.754	present
67662	*P. crucifer*	0.757	*0.307*	absent
65062	*P. phoxocephalus*	0.772	0.494	absent
41121	***P. romeroae* sp. nov.**	0.828	*0.317*	present
30954	***P. limoncochensis***	0.831	*0.294*	present
71457	***P. nankints* sp. nov.**	0.839	*0.314*	present
73812	***P. enigmaticus***	1.044	0.500	present
56418	***P. acuminatus***	1.060	*0.293*	present

**Figure 5.**
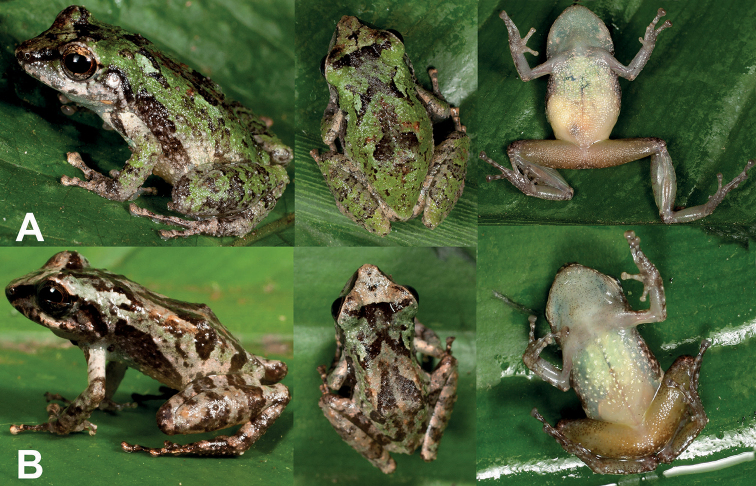
Live adult individuals **A** holotype of *Pristimantis amaguanae* sp. nov., adult female, QCAZ 39274 (SVL = 20.4 mm) **B** paratype of *Pristimantis amaguanae* sp. nov., adult male, QCAZ 39275 (SVL = 16.3 mm). Photographs by Jorge Valencia.

### Systematic accounts

#### *Pristimantis lacrimosus* species group

**Content.** We present a new definition of the group based in our phylogeny and recent taxonomic reviews. We define the *P. lacrimosus* species group to include all species descendant of the most recent common ancestor of *P. eremitus* and *P. lacrimosus* (Fig. 3). Our definition is based on our phylogeny and the data presented by Arteaga et al. (2013), Padial et al. (2014), Ortega-Andrade et al. (2015), Rivera-Correa and Daza (2016), Shepack et al. (2016), Gonzalez-Duran et al. (2017), and Ospina-Sarria and Duellman (2019). We exclude *P. apiculatus* from this group based on its close resemblance to *P. calcarulatus* (Lynch and Duellman 1997), a species that is not closely related to the *P. lacrimosus* species group (e.g., Pyron 2014). We exclude from the group *P. eugeniae* based on the phylogeny by Carrión (2020) which shows that it belongs to the sister clade of the *P. lacrimosus* species group. Ospina-Sarria and Duellman (2019) indicated that *P. sneiderni* is most similar to *P. schultei* and *P. deyi*, two members of the *P. lacrimosus* species group. However, they also stated a resemblance to *P. boulengeri*, a species belonging to the *P. boulengeri* species group. Given this inconsistency we refrain from assigning *P. sneiderni* to the *P. lacrimosus* group until genetic evidence is available. We suspect *P. sneiderni* is not a member of the *P. lacrimosus* species group because it inhabits paramo, a habitat type on which the *P. lacrimosus* species group is absent. Most species of the *P. lacrimosus* species group inhabit foothill Andean forest or lowland tropical rain forest.

The group contains 36 described species: *P. acuminatus* (Shreve 1935), *P. amaguanae* sp. nov. (herein), *P. aureolineatus* (Guayasamin et al. 2006), *P. bromeliaceus* (Lynch 1979), *P. calima*Ospina-Sarria and Duellman 2019, *P. crucifer* (Boulenger 1899), *P. deyi*Lehr et al. 2013, *P. ecuadorensis*Guayasamin et al. 2017, *P. enigmaticus* (Ortega-Andrade et al. 2015), *P. eremitus* (Lynch 1980), *P. galdi* (Jiménez de la Espada 1870), *P. jorgevelosai* (Lynch 1994), *P. lacrimosus* (Jiménez de la Espada 1875), *P. latericius*Batallas and Brito 2014, *P. limoncochensis*Ortega-Andrade et al. 2015, *P. mendax* (Duellman 1978), *P. mindo*Arteaga et al. 2013, *P. moro* (Savage 1965), *P. nankints* sp. nov. (herein), *P. nyctophylax* (Lynch 1976), *P. olivaceus* (Köhler et al. 1998), *P. omeviridis*Ortega-Andrade et al. 2015, *P. ornatissimus* (Despax 1911), *P. padiali*Moravec et al. 2010, *P. pardalinus*, (Lehr et al. 2006), *P. petersi* (Lynch and Duellman 1980), *P. pluvialis*Shepack et al. 2016, *P. royi* (Morales 2007), *P. pseudoacuminatus* (Shreve 1935), *P. romeroae* sp. nov. (herein), *P. schultei* (Duellman 1990), *P. subsigillatus* (Boulenger 1902), *P. tantanti* (Lehr et al. 2007), *P. tayrona* (Lynch and Ruíz-Carranza 1985), *P. waoranii* (McCracken et al. 2007), and *P. zimmermanae* (Heyer and Hardy 1991).

##### Pristimantis amaguanae sp. nov.

Taxon classificationAnimalia

127E3FDA-BD42-5497-8405-41046CFA8CE5

http://zoobank.org/EE43D069-D9E5-456F-A026-3B783EFB2146

Figures 5 , 6 , 7

###### Material.

**Figure 6.**
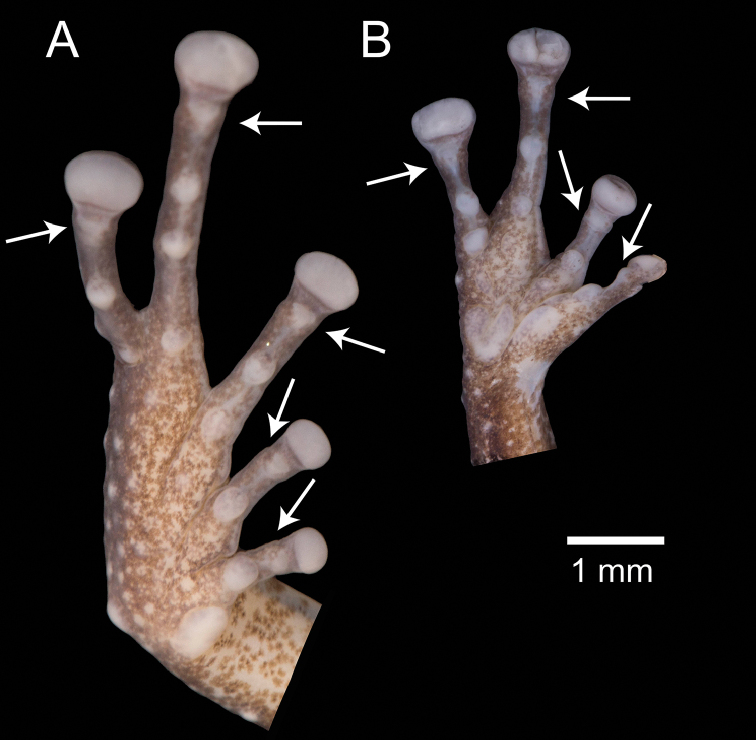
Ventral views of the left hand and foot of *Pristimantis amaguanae* sp. nov. Holotype (QCAZ 39274). Hyperdistal subarticular tubercles are pointed with arrows. Photographs by Julio C. Carrión.

**Figure 7.**
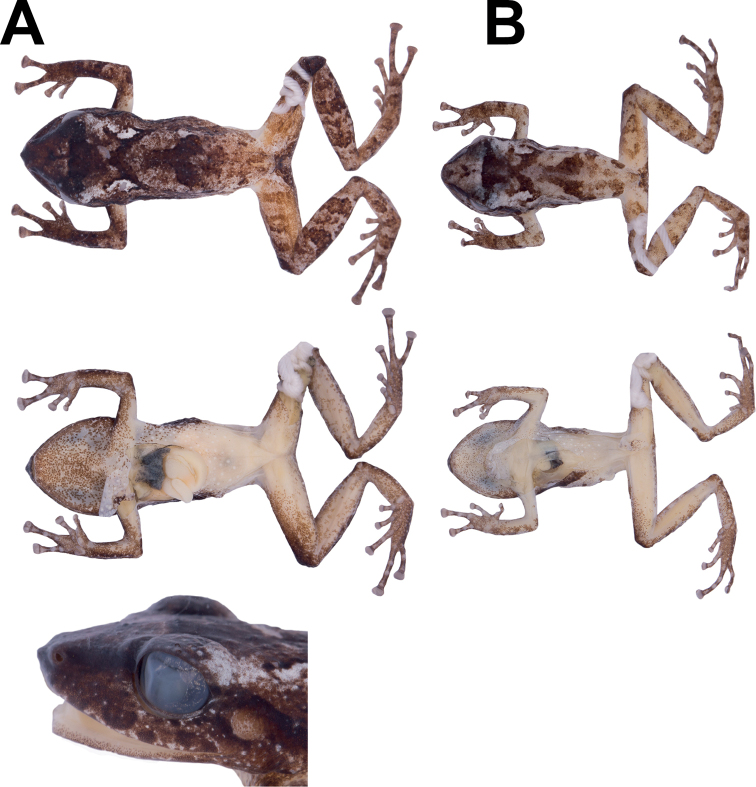
Variation in preserved specimens of *Pristimantis amaguanae* sp. nov. **A** adult female, QCAZ 39274 (SVL = 20.4 mm) **B** adult male, QCAZ 39275 (SVL = 16.3 mm). Photographs by Maricela Rivera.

***Holotype.***QCAZ 39274 (field no. VH 1105; Figs 5–7), adult female from Ecuador, Provincia Pastaza. Surroundings of Villano, AGIP oil camp, K10, Unit 3. (1.4727°S, 77.5359°W), 430 m above sea level, collected by Edwin Carrillo, Galo Díaz, Yadira Mena and Fernando Ayala on 12 October 2008. **Paratype (1).**QCAZ 39275, adult male collected in amplexus with the holotype.

###### Suggested common name.

English: Amaguaña’s Rain Frog. Spanish: Cutín de Amaguaña.

###### Diagnosis.

**Figure 8.**
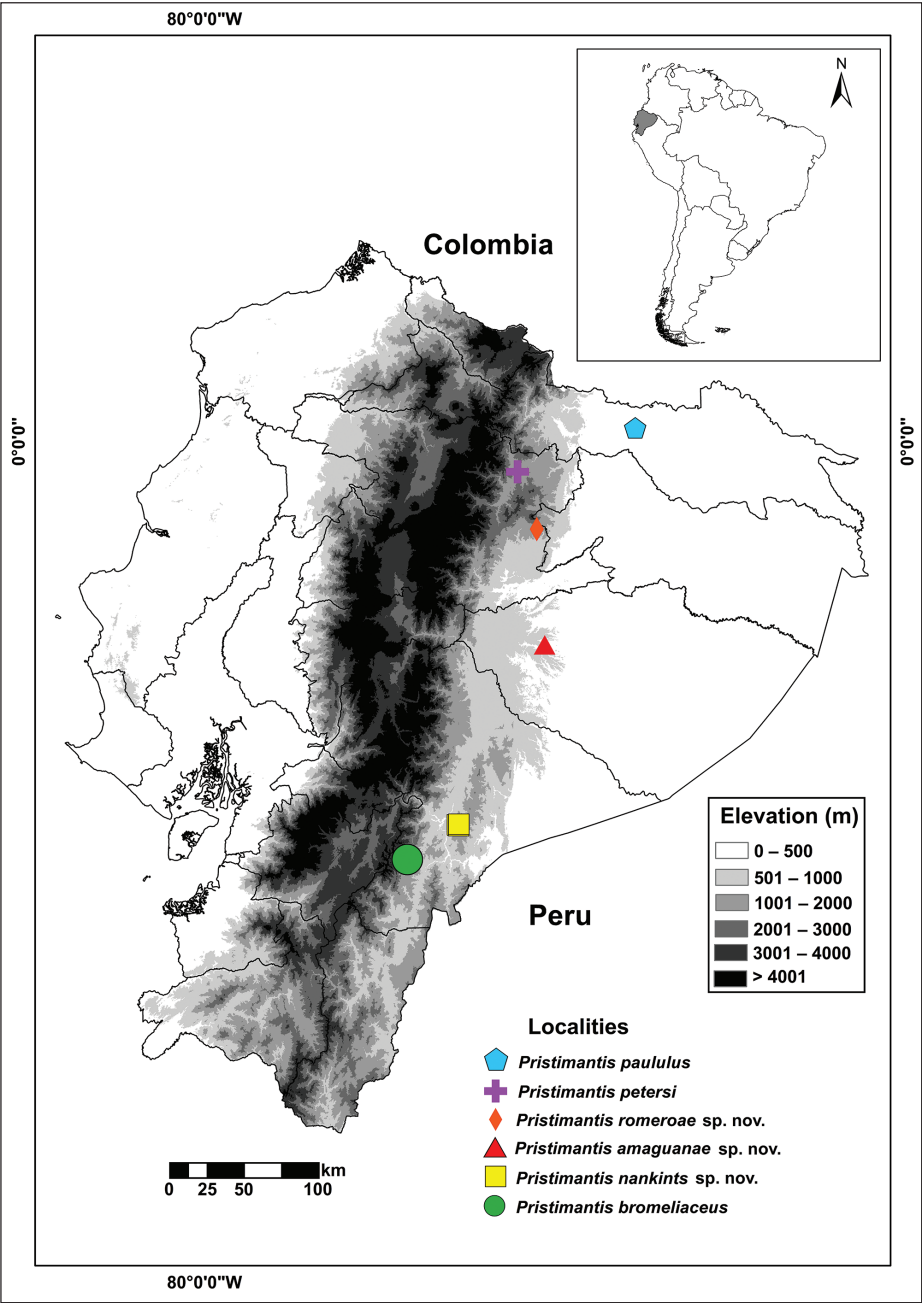
Known distribution of the three new species and type localities of *P. paululus*, *P. petersi*, and *P. bromeliaceus*. Localities are based on specimens deposited at Museo de Zoología of Pontificia Universidad Católica del Ecuador and from Lynch (1974), Lynch (1979), and Lynch and Duellman (1990).

A species of *Pristimantis* characterized by the following combination of characters: (1) skin on dorsum shagreen with conical tubercles, skin on venter areolate with light green warts on the chest; discoidal fold absent; dorsolateral folds absent (Fig. 5); (2) tympanic membrane and tympanic annulus present, its dorsoposterior border converges with supratympanic fold; (3) snout acuminate in dorsal, protruding in lateral profile, with rostral papilla; (4) upper eyelid with several small conical tubercles; cranial crests absent; (5) dentigerous processes of vomers absent; (6) male having vocal slits, nuptial pads absent; (7) finger I shorter than finger II; discs of digits expanded, rounded (Fig. 6); (8) fingers with lateral fringes; hyperdistal subarticular tubercles present; (9) ulnar tubercles present, low and rounded; (10) heel bearing conical tubercles varying from prominent to inconspicuous; inner tarsal fold absent; (11) inner metatarsal tubercle ovoid, elevated, five times the size of round outer metatarsal tubercle; supernumerary plantar tubercles present; (12) toes with narrow lateral fringes; basal toe webbing absent; toe V much longer than toe III (disc on toe III reaches the proximal border of the penultimate subarticular tubercle on toe IV, disc on toe V reaches the distal subarticular tubercle on toe IV); hyperdistal subarticular tubercles present; toe discs as large as those on fingers (Fig. 6); (13) in life, dorsal surfaces of body and limbs olive green or olive brown with black markings; canthal stripe and supratympanic fold black; lips cream with black bars; flanks cream with one broad oblique black bar; chest light green with greenish cream warts; belly yellowish white; iris bronze to reddish copper with black reticulations (Fig. 5); (14) SVL in adult female 20.4 mm (*n* = 1), in adult male 16.3 mm (*n* = 1).

###### Comparison with other species.

In this section, coloration refers to live individuals unless otherwise noticed. The coloration of *Pristimantis amaguanae* resembles that of *P. bromeliaceus* and *P. petersi* (Fig. 5). *Pristimantis amaguanae* can be easily recognized by the presence of transversal dark bands in the hindlimbs (absent or faint in *P. bromeliaceus* and *P. petersi*) and the absence of discoidal fold (present in *P. bromeliaceus* and *P. petersi*). *Pristimantis amaguanae* further differs from *P. bromeliaceus* by its smaller adult size (female SVL = 20.4 mm, males SVL = 16.3 mm vs. *P. bromeliaceus* female SVL = 23.0–28.5 mm, male SVL = 16.7–22.8 mm; Lynch and Duellman 1980). Another small, green *Pristimantis* from the Amazon basin is *P. paululus*. The new species differs by having more tuberculate dorsal skin, discoidal folds absent (folds prominent in *P. paululus*, Lynch 1974), and scattered enlarged light green warts in the venter (small white points in *P. paululus*; Lynch 1974). *Pristimantis pseudoacuminatus* differs by having a truncate snout in profile (acuminate in *P. amaguanae*, Fig. 7) and by having a lighter and more uniform coloration in preservative.

###### Description of the holotype.

Adult female (QCAZ 39274). Measurements (in mm): SVL 20.4; tibia length 9.5; foot length 8.7; head length 8.7; head width 7.7; eye diameter 2.8; tympanum diameter 1.1; interorbital distance 2.5; upper eyelid width 1.9; internarial distance 2.0; eye-nostril distance 2.3; tympanum-eye distance 0.8. Body slender; head slightly longer than wide, wider than body; snout acuminate in dorsal view, protruding in lateral profile, with rostral papilla; canthus rostralis distinct, curved in dorsal view; loreal region concave; interorbital space flat, no cranial crests; eye large, protuberant; upper eyelid bearing numerous small tubercles; tympanic membrane and annulus distinct, rounded in shape, with supratympanic fold partially covering upper and posterodorsal edges; choanae large, rounded, not concealed by palatal shelf of maxillary arc; dentigerous processes of vomers absent; tongue elliptical, posterior border notched, one-third not adherent to floor of mouth.

Skin on dorsum shagreen with scattered tubercles; dorsolateral folds absent; skin on lower flanks and belly areolate with scattered tubercles; skin on throat and chest smooth; discoidal fold absent; skin in upper cloacal region shagreen, wrinkled ventrally, with several tubercles below the cloacal sheath. Forearms slender; conical ulnar tubercles present along outer edge of forearm; all digits bearing pads and discs, broadly expanded and rounded but those of fingers II–IV clearly larger than that on thumb; fingers bearing narrow lateral fringes; relative lengths of fingers I < II < IV < III; subarticular tubercles single, well defined, round in ventral and lateral view; hyperdistal subarticular tubercles present in all fingers; several supernumerary tubercles at base of fingers present, distinct; palmar tubercle bifid, approximately 1.5 size of ovoid thenar tubercle (Fig. 6).

Hindlimbs slender; upper surfaces of hindlimbs smooth; posterior surfaces of thighs smooth, ventral surfaces of thighs slightly areolate; heel bearing low conical tubercles; inner surface of tarsus bearing small, low tubercles; toes with lateral fringes; webbing between toes absent; discs on toes expanded, elliptical, as large as those on fingers; all toes having pads surrounded by circumferential grooves; relative lengths of toes: I < II < III < V < IV; subarticular tubercles rounded, simple; hyperdistal subarticular tubercles present; plantar surface with supernumerary tubercles; inner metatarsal tubercle prominent, ovoid approximately five times of rounded outer metatarsal tubercle (Fig. 6).

*Color of holotype in preservative.* (Fig. 7) Background color pale brown with a dark brown interorbital bar and chevron marks in the scapular and sacrum region; a white mark extending from the posterior border of the upper eyelid to the scapular region; canthal and supratympanic stripe black, extending as a post-axial stripe on lower flanks; a dark brown Y-shaped mark at the tip of snout; dark brown transversal bars on dorsal surfaces of the limbs (three on the forearm, four to five on the thigh, five on the shank, and four on the foot); anal triangle dark brown; flanks and hidden surfaces of thighs pale brown; venter cream with white tubercles; scattered brown flecks on the neck, chest, and lips; ventral surfaces of hindlimbs and forelimbs creamy white with a brown suffusion.

*Color of holotype in life.* (Fig. 5) Dorsal surfaces of body and limbs olive green with black markings; canthal stripe and supratympanic fold black; flanks cream with one broad oblique bar; chest light green with small white spots; belly yellowish white; ventral surfaces of forelimbs and shanks faint green wash; ventral surfaces of thighs pale brown; iris bronze with black reticulations.

###### Variation.

In this section, variation refers to a preserved male QCAZ 39275 (Fig. 7) collected with the holotype in amplexus. The adult male (SVL = 16.3 mm) is smaller than the single known female (SVL = 20.4 mm). Measurements (in mm): tibia length 8.4; foot length 7.1; head length 6.4; head width 5.7; eye diameter 2.1; tympanum diameter 0.8; interorbital distance 2.1; upper eyelid width 1.7; internarial distance 1.7; eye-nostril distance 2.2; tympanum-eye distance 0.5. Male having vocal slits; nuptial pads absent.

*Color in life* (based on digital photographs; Fig. 5). Background coloration is olive brown with faint green dorsolaterally. Marks on dorsum and flanks are similar to the holotype, except for the interorbital bar that is interconnected with the chevron mark in the scapular region. Iris is reddish copper.

###### Distribution, natural history, and conservation status.

This species is only known from the type locality in Provincia de Pastaza, Ecuador at 430 m above sea level (Fig. 8). Natural region is Amazonian Tropical Rainforest (as defined by Ron et al. 2019). The forest is characterized by a high canopy (up to 30 m) with emergent trees that can reach 40 m. Annual precipitation is above 3000 mm and seasonality is low. The amplectant pair was on a leaf 0.4 m above the ground in primary forest near a stream at night.

We recommend assigning *Pristimantis amaguanae* to the Endangered Red List category according to the B2ab(iii) criteria (based on IUCN 2017 guidelines) because it is known from a single locality, its Area of Occupancy is less than 500 km^2^ and its only known locality is at a distance of 1.5 km from deforested areas (based on Google Earth satellite images dating from 2017). A road was built in the area ca. five years ago. Road building is the main predictor of forest destruction in the Ecuadorian Amazon (Sierra 2013).

###### Etymology.

The specific name *amaguanae* is a noun in the genitive case and is a patronym for Tránsito Amaguaña, a leading female figure of the indigenous movement in Ecuador. In 1930 she helped to form the first indigenous organization in Ecuador and during all her life she fought for equality and justice for Ecuadorian poor people.

##### Pristimantis nankints sp. nov.

Taxon classificationAnimalia

40491092-2521-5484-A534-AF5D679AA68D

http://zoobank.org/F3D2457F-6AB8-49F7-9608-1A24A4054782

**Figure 9.**
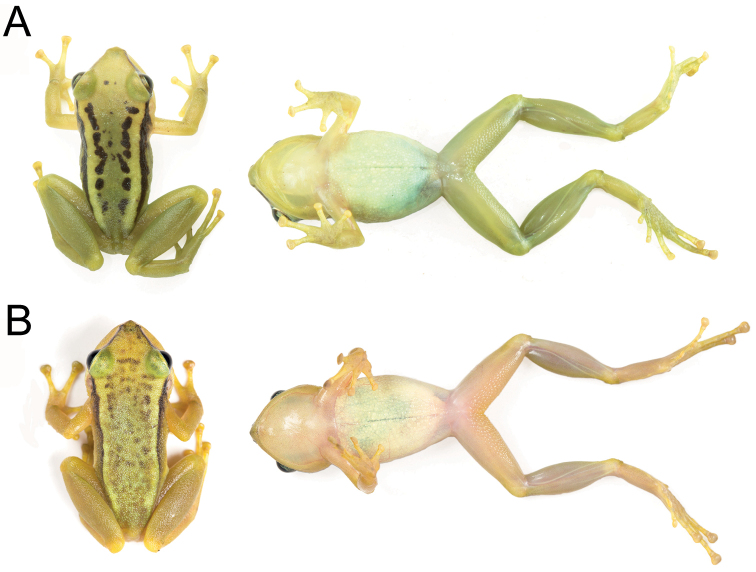
Live individuals of *Pristimantis nankints***A** paratype, subadult male QCAZ 69137 (SVL = 19.6 mm) **B** holotype, adult female QCAZ 71457 (SVL = 30.9 mm). Photographs by Darwin Nuñez (**A**), Gustavo Pazmiño (**B**).

**Figure 10.**
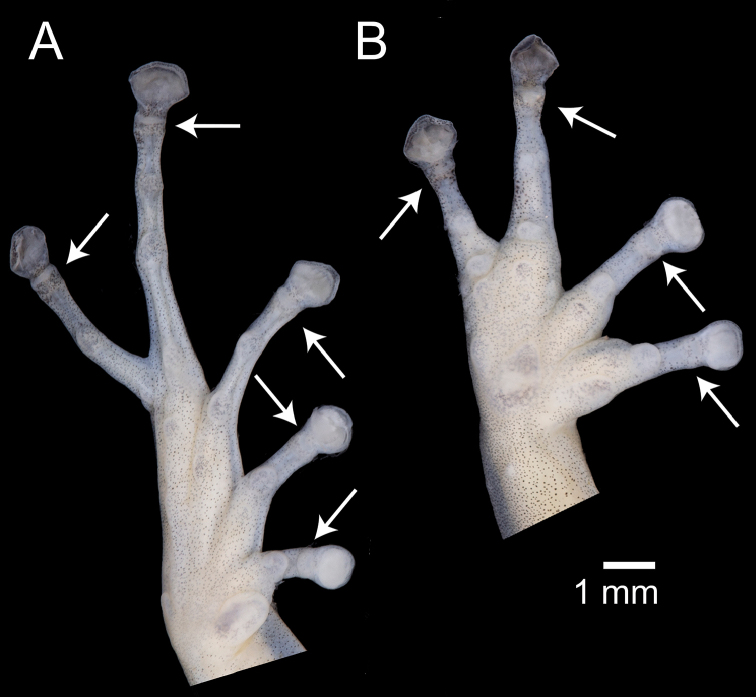
Ventral views of the left hand and foot of *Pristimantis nankints* sp. nov. Holotype (QCAZ 71457). Hyperdistal subarticular tubercles are pointed with arrows. Photographs by Julio C. Carrión.

**Figure 11.**
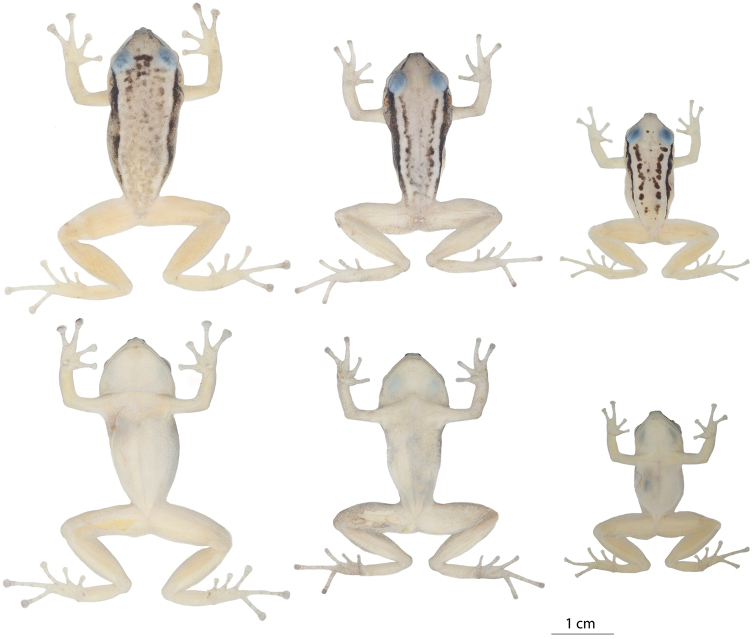
Variation in preserved specimens of *Pristimantis nankints* sp. nov. From left to right, first and second rows: QCAZ 71457 (holotype, adult female, SVL = 30.9 mm), QCAZ 71458 (adult female, SVL = 27.8 mm), QCAZ 69137 (adult male, SVL = 19.6 mm). Photographs by Julio C. Carrión.

**Figure 12.**
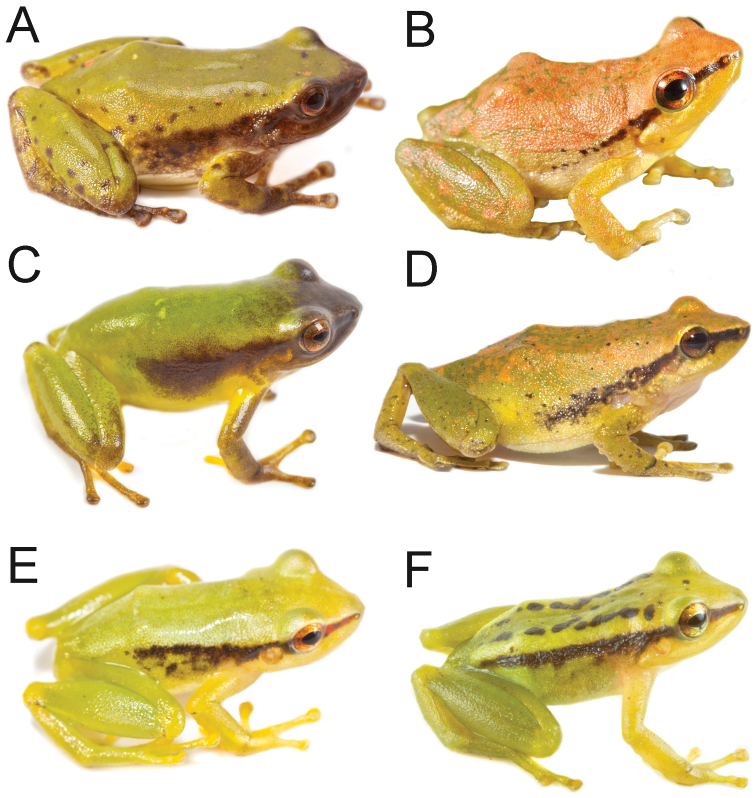
Live adult individuals of the clade of green *Pristimantis* within the *P. lacrimosus* complex and their closest relatives **A***Pristimantis acuminatus*, QCAZ 53845 **B***P. limoncochensis*, QCAZ 37277 **C***P. enigmaticus*, QCAZ 66863 **D***P. omeviridis*, QCAZ 55392 **E***P.* sp., QCAZ 58956 **F***P. nankints* sp. nov. QCAZ 69137 (SVL = 19.6 mm). Photographs by Santiago R. Ron **(A–B)**, Gustavo Pazmiño **(C)**, Diego Quirola **(D)**, Juan Carlos Sánchez **(E)** and Darwin Nuñez **(F).**

Figures 9 , 10 , 11 , 12

###### Material.

***Holotype*.**QCAZ 71457 (field no. SC-PUCE 61965; Figs 9–11), adult female from Ecuador, Provincia Morona Santiago, Cantón Santiago de Méndez. Low part of the Cutucú mountain range in the vicinity of the house of Mr. Carlos Hurtado (2.78325°S, 78.15878°W), 1413 m above sea level. Collected by Diego Almeida, Diego Paucar, Darwin Núñez, Eloy Nusirquia and Ricardo Gavilanes on 01 January 2018. **Paratypes (2).** All specimens were collected in Ecuador, Provincia Morona Santiago. QCAZ 71458 (field no. SC-PUCE 61965), adult female collected with the holotype. QCAZ 69137 (field no. SC-PUCE 60012); adult male from Ecuador, Cantón Santiago, Puchimi (2.7780°S, 78.1682°W), 1364 m above sea level, collected by Diego Almeida, Darwin Núñez, Eloy Nusirquia and Jefferson Mora on 14 September 2017.

###### Suggested common name.

English: Nankints Rain Frog. Spanish: Cutín de Nankints.

###### Diagnosis.

**Figure 13.**
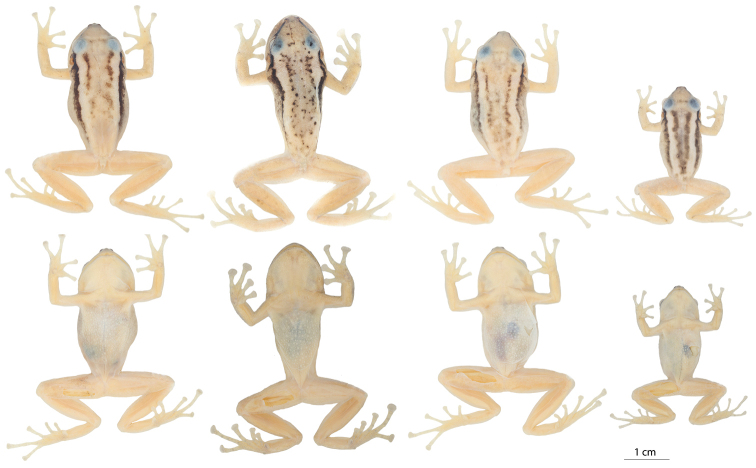
Variation in preserved specimens of *Pristimantis romeroae* sp. nov. From left to right, first and second rows: QCAZ 41121 (holotype, SVL = 31.1 mm, adult female), QCAZ 41103 (SVL = 33.8 mm, adult female), QCAZ 41128 (SVL = 31.15 mm, adult female), QCAZ 41222 (SVL = 23.8 mm, adult male). All specimens are shown at the same scale. Photographs by Julio C. Carrión.

A species of *Pristimantis* characterized by the following combination of characters: (1) skin on dorsum shagreen, skin on venter areolate with scattered warts, smooth on throat; discoidal fold absent; dorsolateral folds absent; (2) tympanic membrane and tympanic annulus present, upper edge of tympanic annulus covered by supratympanic fold; (3) snout short, protruding in lateral profile, acuminate in dorsal view, with rostral papilla; (4) upper eyelid without conical tubercles; cranial crests absent; (5) dentigerous processes of vomers present, prominent, oblique; (6) male having vocal slits, nuptial pads absent; (7) finger I shorter than finger II; discs of digits moderately expanded, rounded; (8) fingers bearing narrow lateral fringes; hyperdistal subarticular tubercles present; (9) ulnar and tarsal tubercles present, those on the tarsus are flattened and low, nearly inconspicuous; (10) heel without conical tubercles; inner tarsal fold present; (11) inner metatarsal tubercle prominent, elliptical, approximately 3× as large as rounded, conical outer metatarsal tubercle; outer metatarsal tubercle small, rounded; supernumerary plantar tubercles inconspicuous (Fig. 10); (12) toes bearing narrow lateral fringes; toe webbing absent except for basal webbing between toes III and IV; toe V much longer than toe III (disc on toe III extends to the distal edge of the medial subarticular tubercle on toe IV, disc on toe V extends beyond the proximal edge of the distal subarticular tubercle on toe IV); hyperdistal subarticular tubercles present; toe discs smaller than those on fingers (Fig. 10); (13) in life, dorsum lime green to olive green with black to brown marks on dorsum; flanks green with black to brown longitudinal stripes on upper edge; chest and belly greenish cream, throat yellowish green; ventral surface of thighs lime green to pinkish green. Iris bronze with a broad dark brown horizontal band and black reticulations; (14) SVL in adult females 29.4 mm ± 2.2 (27.8–30.9, n = 2), SVL in one adult male 19.6 mm.

###### Comparisons with other species

(Fig. 12). In this section, coloration refers to live individuals unless otherwise noticed. *Pristimantis nankints* and *P. romeroae* have similar coloration in preservative. *Pristimantis nankints* differs by having small warts in the venter (numerous large warts in *P. romeroae*) and by the size and shape of discs on fingers: expanded and truncate in *P. romeroae* vs. moderately expanded and rounded in *P. nankints*. Its green dorsal coloration resembles that of *P. acuminatus*, *P. enigmaticus*, *P. limoncochensis*, *P. omeviridis*, *P. pseudoacuminatus*, and *P. tantanti*. It differs from all of them by having a dark stripe bordering the upper edge of the flanks (dark stripe absent or if present, it is an oblique-lateral stripe starting behind the eye and ending near the ventral edge of the flank at midbody). It further differs from *P. acuminatus*, *P. limoncochensis*, and *P. tantanti* by having a prominent tympanum (tympanum absent in the three species; Duellman and Lehr 2009). *Pristimantis enigmaticus* and *P. omeviridis* differ by having a smaller tympanum (12–13% of head length [Ortega-Andrade et al. 2015] vs. 20–25% in *P. nankints*). *Pristimantis pseudoacuminatus* differs by having sparse tubercles and warts on the dorsum (tubercles and warts absent in *P. nankints*; Shreve 1935).

###### Description of the holotype.

Adult female (QCAZ 71457; Figs 9–11). Measurements (in mm): SVL 30.9; tibia length 13.2; foot length 14.9; head length 11.4; head width 10.6; eye diameter 3.0; tympanum diameter 2.3; interorbital distance 3.8; upper eyelid width 2.8; internarial distance 2.3; eye-nostril distance 3.7; tympanum-eye distance#1.7. Body slender; head slightly wider than long, wider than body; snout short, protruding in lateral profile, acuminate in dorsal view, with rostral papilla; canthus rostralis distinct, slightly curved in dorsal view; loreal region slightly concave; interorbital space flat, no cranial crests; upper eyelid ca. 75% of interorbital distance; lacking tubercles, no interocular fold. Tympanic membrane and annulus present, rounded in shape, with supratympanic fold covering upper edge; horizontal diameter of tympanum ca. 75% of eye diameter, separated from eye by a distance of a complete tympanum length; choanae large, rounded, not concealed by palatal shelf of maxillary arc; dentigerous processes of vomers present, prominent, oblique, bearing 5 teeth, tongue cordiform.

Skin on dorsum shagreen; dorsolateral folds absent; skin on belly and posterior half of chest areolate with scattered warts; skin on anterior half of chest and throat smooth; discoidal fold absent; skin in upper cloacal region shagreen. Forearms slender with a row of four conical ulnar tubercles in outer edge of forearm; fingers large and slender, all with round discs; fingers bearing narrow lateral fringes; relative lengths of fingers I < II < IV < III; subarticular tubercles single, well defined, round in ventral view; hyperdistal subarticular tubercles present in all fingers; supernumerary palmar tubercles present, distinct; palmar tubercle bifid, 2× size of ovoid thenar tubercle (Fig. 10).

Hindlimbs slender; tibia length ca. 50% of SVL; upper surfaces of hindlimbs smooth; foot length ca. 46 % of SVL, posterior surfaces of thighs smooth, ventral surfaces of thighs slightly areolate; knee and heel lacking tubercles; inner surface of tarsus lacking tubercles; toes bearing narrow lateral fringes; webbing between III and IV toes present at the base; discs on toes broadly expanded, rounded, relatively smaller than fingers; all toes having pads surrounded by circumferential grooves; relative lengths of toes: I < II < III < V < IV; subarticular tubercles rounded; hyperdistal subarticular tubercles present in all toes; plantar surface with inconspicuous supernumerary tubercles; inner metatarsal tubercle prominent, elliptical, approximately three times as large as rounded, conical outer metatarsal tubercle (Fig. 10).

*Color of holotype in preservative.* (Fig. 11) Background color pale gray with dark brown interorbital bar; light canthal and supratympanic black stripe continued by a long dorsolateral stripe bordering the upper flank; dorsal brown marks scattered on all dorsum except for two longitudinal bands adjacent to the dark dorsolateral stripe; color of venter, chest, and ventral surfaces of the limbs pale cream.

*Color of holotype in life.* (Fig. 9) Dorsal surfaces dark green with black spots and limbs yellowish green; canthal stripe and supratympanic fold black, continued by black dorsolateral stripe bordering the upper flank, the black stripe gradually fades in the upper flank and limits dorsally with a parallel light green band; chest and belly greenish cream, throat yellowish cream; ventral surfaces of forelimbs yellowish green and shanks faint green; ventral surfaces of thighs pinkish green; iris bronze with a broad dark brown horizontal band and black reticulations.

###### Variation.

(Fig. 11) In this section, coloration refers to preserved individuals. In the type series, the adult male has an SVL = 19.6 mm and the adult female 27.8 mm; (Table 3). The male has vocals slits and lacks nuptial pads. Dorsal coloration in preservative is light gray (e.g., QCAZ 71458) to yellowish cream (e.g., QCAZ 69137) with black dorsolateral bars and a black canthal stripe. Both paratypes have a lighter dorsolateral band bordered by the dark dorsolateral stripe and delimited medially by a faint black stripe (QCAZ 71458) or a row of black marks (QCAZ 69137). Both paratypes lack an interorbital bar but QCAZ 69137 has two dark marks instead. In QCAZ 69137 the flanks are yellowish cream; in QCAZ 71458 the flanks are gradually darker towards the black dorsolateral stripe. The tips of the fingers have the same color as the rest of the finger in QCAZ 69137 while in QCAZ 71458 they are darker.

**Table 3.** Morphometric variables of *Pristimantis nankints* sp. nov. and *Pristimantis romeroae* sp. nov. Mean ± SD is given with range in parentheses. All measurements are in millimeters.

**Variable**	***P. nankints* sp. nov.**		***P. romeroae* sp. nov.**	
	male	female	male	female
	n = 1	n = 2	n = 1	n = 3
Snout-vent length	19.6	29.4 ± 2.2 (27.8–30.9)	23.8	32.0 ± 1.6 (31.1–33.8)
Tibia length	10.4	13.3 ± 0.2 (13.2–13.5)	9.9	14.6 ± 0.6 (14.2–15.2)
Foot length	10.8	14.5 ± 0.6 (14.1–14.9)	11.1	15.8 ± 0.3 (15.6–16.1)
Head length	7.0	10.7 ± 1.0 (10.0–11.4)	8.2	11.4 ± 0.6 (10.8–12.0)
Head width	6.3	10.3 ± 0.4 (10.0–10.6)	7.7	11.3 ± 0.4 (11.0–11.7)
Eye diameter	2.6	2.8 ± 0.2 (2.7–3.0)	2.8	3.2 ± 0.3 (2.9–3.4)
Tympanum diameter	1.8	2.3 ± 0.1 (2.3–2.4)	2.3	2.8 ± 0.5 (2.4–3.3)

*Color in life* (based on digital photographs of an adult male QCAZ 69137) (Fig. 9): dorsal surfaces are lime green; black-reddish canthal stripe is continued by black supratympanic fold and black dorsolateral stripe bordering the upper flank, the black stripe limits dorsally with a parallel light lime green band limited medially by two rows of black round marks; flanks, ventral and dorsal surfaces of limbs are green; belly and chest are greenish cream, throat greenish yellow; fingers, and toes greenish yellow; iris bronze.

###### Distribution, natural history, and conservation status.

*Pristimantis nankints* has been recorded at one locality in the eastern Andean slopes of Ecuador, Provincia Morona Santiago, Cordillera del Cutucú, 1364–1413 m above sea level (Fig. 8). Natural Region is Andean Eastern Montane Forest (according to Ron et al. 2019 classification) which is characterized by evergreen trees covered by mosses and abundant epiphytic plants.

Specimens were found at night along crystalline creek surrounded by secondary forest. The holotype was perching on a leaf 2 m above the ground, next to the stream; after capture, while in the plastic bag, the female deposited 22 eggs. The adult male was collected on secondary forest, on a terrestrial bromeliad. The female paratype was perching on a leaf 30 cm above the ground.

*Pristimantis nankints* distribution area is a mosaic of forest and deforested areas (based on Ministerio de Ambiente del Ecuador 2013). Its occurrence in secondary forest, near artificial open areas, indicates at least some level of resilience to anthropogenic habitat change. Nevertheless, there is not enough information to assess its risk of extinction. Therefore, we recommend its assignment to the Data Deficient Red List Category (DD) (based on IUCN 2017 guidelines).

###### Etymology.

The species name is a noun in apposition that refers to Nankints, a small hamlet in Cordillera del Cóndor, Ecuador, that used to be inhabited by Shuar native Americans. Its dwellers were violently evicted and Nankints was destroyed in 2016 to establish a mining camp. Large scale mining projects generate widespread deforestation and pollution in the Andes. The species name is a tribute to Ecuadorian people who have resisted mining activities in defense of the environment. *Nankints* is a shuar word that means spear.

##### Pristimantis romeroae sp. nov.

Taxon classificationAnimalia

DAC85181-74C9-5295-8F29-DA99AC0A1B35

http://zoobank.org/41809854-F7F5-4D0D-B023-97425E666D0C

Figures 1 , 13 , 14

###### Material.

**Figure 14.**
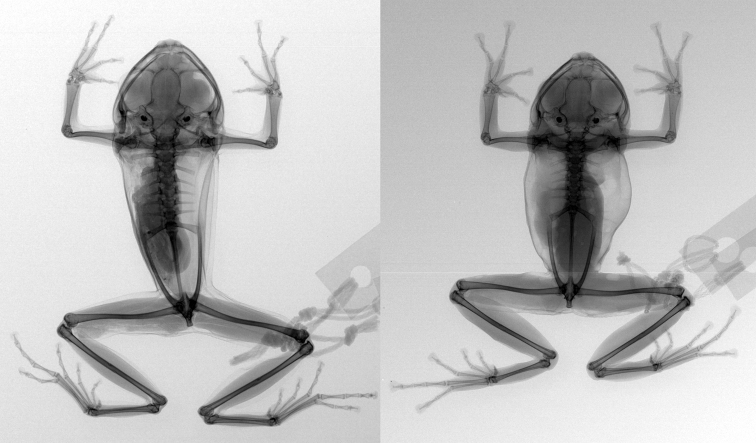
X-rays of *Pristimantis nankints* sp. nov. and *Pristimantis romeroae* sp. nov. Left, *Pristimantis nankints* sp. nov. holotype, QCAZ 71457; right, *Pristimantis romeroae* sp. nov. holotype, QCAZ 41121.

***Holotype*.** (Figs 1, 13, 14) QCAZ 41121 (field no. SC-PUCE 27602), adult female from Ecuador, Provincia de Napo, Cantón Archidona, Parroquia Cotundo, Pacto Sumaco-Volcán Sumaco road, El Mirador cottage, 3 km from the cottage to the volcano on Río Pucuno, SSE slope of the Sumaco volcano, 10 km airline distance from the summit (0.633915°S, 77.59228°W), 1602 m above sea level, collected by Elicio Tapia and Raúl E. Ruiz on 21 March 2009.

***Paratypes* (3)**. Provincia de Napo: QCAZ 41103, 41128 adult females, QCAZ 41122 adult male. Collected at the type locality with the holotype by Elicio Tapia and Raúl E. Ruiz on 21 March 2009.

###### Suggested common name.

English: Romero’s Rain Frog. Spanish: Cutín de Romero.

###### Diagnosis.

A species of *Pristimantis* characterized by the following combination of characters: (1) skin on dorsum shagreen, skin on venter areolate with scattered warts; discoidal fold absent; dorsolateral folds absent; (2) tympanic membrane and tympanic annulus present, upper edge of tympanic annulus covered by supratympanic fold; (3) snout short, truncate in dorsal view, slightly protruding in lateral profile, with small rostral papilla; (4) upper eyelid with several small tubercles; cranial crests absent; (5) dentigerous processes of vomers present, prominent, moderately oblique; (6) male having vocal slits, nuptial pads present on finger I; (7) finger I slightly shorter than finger II; discs of digits expanded, truncate; (8) fingers with lateral fringes; hyperdistal subarticular tubercles present; (9) ulnar tubercles absent, tarsal tubercles present, subconical, conspicuous; (10) heel with one, nearly inconspicuous, small subconical tubercle or without tubercles; inner tarsal fold absent; (11) inner metatarsal tubercle prominent, elliptical, approximately three times as large as rounded, conical outer metatarsal tubercle; supernumerary plantar tubercles present (Fig. 1); (12) toes with lateral fringes; toe webbing absent; toe V much longer than toe III (disc on toe III extends to the distal edge of the medial subarticular tubercle on toe IV, disc on toe V extends beyond the proximal edge of the distal subarticular tubercle on toe IV); hyperdistal subarticular tubercles present in all toes; toe discs smaller than those on fingers (Fig. 1); (13) life, coloration unknown; (14) SVL in adult females 31.1–33.8 mm (n = 3), adult male = 23.8 mm (n = 1).

###### Comparisons with other species.

In this section, coloration refers to preserved individuals (Fig. 13). *Pristimantis romeroae* resembles *P. nankints* in coloration. *Pristimantis romeroae* differs by having numerous large warts on the venter (small warts in *P. nankints*) and by the size and shape of discs on fingers: expanded and truncate in *P. romeroae* vs. moderately expanded and rounded in *P. nankints*. Its predominately pale creamy orange dorsal coloration resembles that of preserved *P. acuminatus*, *P. enigmaticus*, *P. limoncochensis*, *P. omeviridis*, *P. pseudoacuminatus*, and *P. tantanti*. It differs from all of them by having a dark stripe bordering the upper edge of the flanks (dark stripe absent or if present, it is an oblique-lateral stripe starting behind the eye and ending near the ventral edge of the flank at midbody). It also differs from *P. acuminatus*, *P. limoncochensis*, and *P. tantanti* by having a conspicuous tympanum (absent in the three species). *Pristimantis romeroae* can be further distinguished from *P. enigmaticus* and *P. omeviridis* by having a larger tympanum (21–27% of head length in *P. romeroae* vs. 12–13% in both species; Ortega-Andrade et al. 2015). *Pristimantis pseudoacuminatus* differs by having sparse tubercles and warts on the dorsum (absent in *P. romeroae*; Shreve 1935).

###### Description of the holotype.

Adult female (QCAZ 41121). Measurements (in mm): SVL 31.1; tibia length 14.3; foot length 15.7; head length 11.2; head width 11.0; eye diameter 3.2; tympanum diameter 2.4; interorbital distance 4.0; upper eyelid width 3.0; internarial distance 3.4; eye-nostril distance 3.2; tympanum-eye distance 0.9. Semi-slender body; head much wider than long, wider than body; snout short, truncate in dorsal view, slightly protruding in lateral profile, with rostral papilla; canthus rostralis distinct, slightly curved in dorsal view; loreal region concave; interorbital space flat, lacking cranial crests; eye large; upper eyelid ca. 73% of interorbital distance; lacking tubercles, no interocular fold. Tympanic membrane and annulus present, rounded in shape, its upper and posterodorsal edges covered by supratympanic fold; horizontal diameter of tympanum ca. 54% of eye diameter, separated from eye by a distance ca. 45% tympanum length; choanae large, elliptical, non-concealed by palatal shelf of maxillary arc; dentigerous processes of vomers present, prominent, moderately oblique, narrowly separated, bearing seven teeth, tongue large, rounded, posterior border notched, 15% not adherent to floor of mouth.

Skin on dorsum and flanks shagreen; dorsolateral folds absent; skin on belly and posterior half of chest areolate with scattered warts; skin on throat and anterior half of chest smooth; discoidal fold absent; skin in upper cloacal region smooth. Forearms slender with three ill-defined, low ulnar tubercles in distal, medial and proximal outer edge of forearm; fingers large and slender, all fingers with pads surrounded by circumferential grooves, truncate discs; bearing narrow lateral fringes; relative lengths of fingers I < II < IV < III; subarticular tubercles single, round in ventral and lateral view; hyperdistal subarticular tubercles present; bearing few, inconspicuous, low supernumerary tubercles, palmar tubercle bifid, twice the size of elliptical thenar tubercle (Fig. 1).

Hindlimbs slender; tibia length ca. 50% of SVL; upper surfaces of hindlimbs smooth; foot length ca. 45 % of SVL, posterior surfaces of thighs shagreen, ventral surfaces of thighs smooth; knee and heel lacking tubercles; inner surface of tarsus lacking tubercles; toes bearing narrow lateral fringes; webbing between toes absent; discs on toes broadly expanded, truncate, the same size than fingers; all toes having pads surrounded by circumferential grooves; relative lengths of toes: I < II < III < V < IV; subarticular tubercles rounded, simple; hyperdistal subarticular tubercles present; plantar surface with numerous indistinct supernumerary tubercles; inner metatarsal tubercle prominent, elliptical, approximately 3 times the size of rounded, conical outer metatarsal tubercle (Fig. 1).

*Color of holotype in preservative.* (Fig. 13) Background color pale creamy orange with faint interorbital line; long, thick, dark brown dorsolateral bars; dorsum with paler blotches clustered in two parallel stripes at the scapular region approximately half the length of the dorsolateral bars; face with dark brown canthal and supratympanic stripes, supratympanic stripe suffused with the dark brown dorsolateral bar; flanks the same back ground color with minute dark spots (visible under magnification) densely distributed; dorsal surfaces of limbs yellowish cream brighter than dorsum with scattered minute dark brown spots visible under magnification; ventral surface of body yellowish cream; plantar and palmar surfaces dirty cream.

*Color of holotype in life.* Unknown but presumably green, similar to its most closely relatives (e.g., *P. nankints*, *P. enigmaticus*) which have a similar clear coloration in preservative (Figs 9 and 12).

###### Variation.

(Fig. 13) In this section, coloration refers to preserved individuals unless otherwise noted. In the type series, the adult male has an SVL = 23.8 mm, lower than the adult female SVL (range 31.1–33.8 mm; Table 3). Males have vocals slits and nuptial pads on finger I. Dorsal coloration is creamy tan (e.g., QCAZ 41121) with a black canthal stripe followed by black dorsolateral stripes. Marks on dorsum vary from scattered dark brown spots (e.g., QCAZ 41103) to two longitudinal brown strips starting behind the head and converging medially in the sacral region (QCAZ 41122) with or without a fine interorbital bar. Flanks are cream; venter and ventral surfaces of limbs vary from creamy white (e.g., QCAZ 41122) to yellowish cream (e.g., QCAZ 41103). The belly has scattered white warts (e.g., QCAZ 41103, 41121).

*Color in life*: unknown but presumably green (see description of the holotype).

###### Distribution, natural history, and conservation status.

*Pristimantis romeroae* is known from one locality at the eastern Andean slopes of Ecuador, Provincia de Napo, on the SSE slope of the Sumaco volcano, 1602 m above sea level (Fig. 8). Natural Region is Andean Eastern Montane Forest (according to Ron et al. 2019 classification) which is characterized by evergreen trees covered by mosses and abundant epiphytic plants. Except for QCAZ 41128, all specimens were found on a spiny bromeliad 6 cm from the ground by the day. QCAZ 41128 was found also by day on a bromeliad of a recently fallen tree.

In 2008, one year before the specimens were collected, the type locality was at a distance of < 1 km from agricultural deforested areas (based on Ministerio de Ambiente del Ecuador 2013) suggesting at least some level of tolerance to habitat degradation. Available information is insufficient to determine the risk of extinction of this species known from a single locality. Lack of records may partly be a consequence of its association with bromeliads which generally grow at heights unreachable during herpetological searches. We suggest to assigning *P. romeroae* to the Data Deficient Red List Category (DD) (based on IUCN 2017 guidelines).

###### Etymology.

The species name is a noun in the genitive case and is a patronym for Giovanna Romero, an Ecuadorian botanist and SRR’s wife. For almost two decades, she has supported SRR’s research in countless ways and this is a long-overdue tribute.

## Discussion

### Subarticular tubercles and phalange morphology in *Pristimantis*

Subarticular tubercles are round dermal protuberances, below the articulations of phalanges, on the underside of the hands and feet of anurans. According to the most recent comprehensive reviews of morphological characters of *Pristimantis* (Lynch and Duellman 1997; Duellman and Lehr 2007), strabomantid frogs have: (1) one subarticular tubercle on fingers I, II and toes I, II, (2) two subarticular tubercles on fingers III, IV and toes III, IV, and (3) three subarticular tubercles on toe IV. While describing the morphology of the new species, we noticed the presence of an additional subarticular tubercle underlying the articulation of the last phalange on each finger and toe (arrows in Fig. 1). Those tubercles are similar in size to other subarticular tubercles. While preparing this publication, we became aware of a recent publication by Ospina-Sarria and Duellman (2019) on which they report the same tubercles and named them “hyperdistal tubercles”. Prior to Ospina-Sarria and Duellman (2019), the only mention we could find of hyperdistal tubercles in Strabomantid frogs was made by Lynch (1999) who stated “in many species there is a tubercle poorly defined that corresponds to the articulation of the terminal phalange (which supports the disk) and the penultimate phalange. This is a subarticular tubercle but because it is not as developed as the others, conventionally we do not take it into account”. Our observations indicate that hyperdistal tubercles can be as large or larger than other subarticular tubercles (e.g., Fig. 1). Variation between presence and absence of hyperdistal tubercles appear to be continuous. In some species, like *P. phoxocephalus*, there is an inconspicuous tubercle at the base of the disk. In others, like *P. katoptroides*, the tubercle is larger than that of *P. phoxocephalus* but smaller than the hyperdistal tubercles observed in the *P. lacrimosus* species group. Future studies on the evolution and functional significance of hyperdistal tubercles would require methodologies to code it as a continuous character.

The available information on the phylogenetic distribution of hyperdistal tubercles and the morphology of the terminal phalanges suggest that they have phylogenetic signal and diagnostic value. Hyperdistal tubercles are present in all examined species of the *P. lacrimosus* species group (ten species: Table 2, Ospina-Sarria and Duellman 2019). They are also present in *P. orcesi* and *P. eugeniae*, two members of the sister clade of the *P. lacrimosus* species group (Table 2, Ospina-Sarria and Duellman 2019). In contrast, they were absent in the distantly related *P. crenunguis*, *P. achatinus*, *P. condor*, and *P. lanthanites* (Table 2). The available evidence suggests that the hyperdistal tubercles may be shared by the *P. lacrimosus* species group and its sister clade. Hyperdistal tubercles were also found in three species belonging to the closely related *P. boulengeri* and *P. leptolophus* species groups: *P. angustilineatus*, *P. boulengeri*, and *P. leptolophus* (Ospina-Sarria and Duellman 2019). They are also present in *P. eriphus*, *P. katoptroides*, and *P. orpacobates*, species that are not closely related to the *P. lacrimosus* species group (Table 2). The distribution of hyperdistal tubercles among *Pristimantis* suggests several independent origins. An exhaustive analysis of the distribution of hyperdistal tubercles on a phylogenetic context is necessary to understand its evolution.

Interestingly, our results suggest that the presence of hyperdistal tubercles is linked to two osteological characters of the terminal phalanges (Table 2). First, species with hyperdistal tubercles tend to have long terminal phalanges (relative to the penultimate phalange; Fig. 2, Table 2). Second, they tend to have narrow T-shaped expansions at the end of the terminal phalange. Our results on 21 species of *Pristimantis* (Table 2) suggest future venues of research to discover morphological synapomorphies for *Pristimantis* clades, a task that has been elusive until now.

The correlation between the presence of hyperdistal tubercles and the morphology of the terminal phalanges has not been tested. We suspect that in species with short ultimate phalanges, like *P. crenunguis* (Fig. 2), the disk pad overlaps the phalange articulation and, therefore, the hyperdistal tubercle. This overlap may explain the absence of hyperdistal tubercles in species with short terminal phalanges (Fig. 2). In species with long terminal phalanges, like *P. romeroae*, the disk pad does not overlap with the articulation and the subarticular tubercle is separate from the disk pad and, therefore, is distinct.

### The *Pristimantis lacrimosus* species group originated in the Chocoan forests

Our reconstruction of ancestral basin indicates that the *P. lacrimosus* species group originated in the Chocoan forests of the Pacific basin of Ecuador and Colombia. This result was unexpected because, by far, most species of the group occur in the Amazon basin (26 described species out of 36). Our reconstruction suggests that the Amazon basin was colonized on a single event. The paucity of colonization events across the Andes demonstrates the pivotal role of the Andean barrier in the diversification of *Pristimantis*. There is a single colonization event from the Amazon Basin to the Chocoan forests with *P. moro*. Because *P. moro* is also distributed in Central America, we suspect that its presence in the Chocoan forest is a result of a colonization from central America instead of colonization across the Andes. The Amazon basin was also the origin of *P. jorgevelosai*, a species distributed in the Magdalena river basin and embedded in an otherwise Amazonian clade (Fig. 4).

Our biogeographic reconstruction suggests that the *P. lacrimosus* species group had higher diversification rates in the Amazon basin relative to the Chocó region. There are two clades inhabiting the Chocoan region. One of them is older than the Amazonian clade and yet it only has four species. The second clade is sister to the Amazonian clade. Although both clades have the same age, the Chocoan clade has only four species compared to 19 species in the Amazonian clade. These differences in diversification rates are inconsistent with the time-for-speciation hypothesis, which predicts the highest richness in first colonized regions (Stephens and Wiens 2003). Additional studies are needed to determine if the higher diversification rate in the Amazon region, relative to the Chocó, observed in the *P. lacrimosus* group is a generality among *Pristimantis* inhabiting both regions.

## Supplementary Material

XML Treatment for Pristimantis amaguanae

XML Treatment for Pristimantis nankints

XML Treatment for Pristimantis romeroae

## Appendix 1

### Examined specimens

*Pristimantis petersi*. ECUADOR: PROVINCIA NAPO: Cantón: Archidona. Cocodrilos, Cocodrilo Station (0.6545°S, 77.7848°W) 1732 m, (QCAZ 31416, 31417); road from Baeza to Archidona (77.7929°W, 0.6711°S) 1575 m, (QCAZ 63452, 63453, 63454, 63455, 63456). PROVINCIA PASTAZA: Cantón Mera, Ankaku Private Station (78.0779°W, 1.2792°S) 2300 m (QCAZ 45846, 45847, 45848, 45849, 45850, 45892, 45898).

*Pristimantis bromeliaceus*. ECUADOR: PROVINCIA MORONA SANTIAGO: Cantón: Gualaquiza. Chinguida (3.2263°S, 78.7105°W) 1930 m (QCAZ 56454); Zuñac (2.1934°S, 78.3597°W) 2371 m, (QCAZ 61836). PROVINCIA ZAMORA CHINCHIPE: Cantón: Nangaritza. Campo Maicu (4.1195°S, 78.5766°W) 1667 m (QCAZ 55469); La Canela (4.5728°S, 78.8915°W) 1736 (QCAZ 63272).

*Pristimantis paululus*. ECUADOR: PROVINCIA NAPO. Jatun Sacha Biological Reserve (1.05°S, 77.6000°W) 388 m (QCAZ 09099); (1.0660°S, 77.6170°W) 405 m, (QCAZ 53002, 53016). PROVINCIA ORELLANA. San José de Payamino (0.4794°S, 77.2945°W) 318 m, (QCAZ 56656). Cantón: Joya de los Sachas. La Parquer (0.34243°S, 76.8665°W) 284 m, (QCAZ 63130).
